# Network modeling of BVD transmission

**DOI:** 10.1186/1297-9716-43-11

**Published:** 2012-02-10

**Authors:** Mark Tinsley, Fraser I Lewis, Franz Brülisauer

**Affiliations:** 1 C. Eugene Bennett Department of Chemistry, West Virginia University, Morgantown, USA; 2 Section of Epidemiology, University of Zurich, Switzerland; 3 SAC Consulting Veterinary Services, Inverness, UK

## Abstract

Endemic diseases of cattle, such as bovine viral diarrhea, have significant impact on production efficiency of food of animal origin with consequences for animal welfare and climate change reduction targets. Many modeling studies focus on the local scale, examining the on-farm dynamics of this infectious disease. However, insight into prevalence and control across a network of farms ultimately requires a network level approach. Here, we implement understanding of infection dynamics, gained through these detailed on-farm modeling studies, to produce a national scale model of bovine viral diarrhea virus transmission. The complex disease epidemiology and on-farm dynamics are approximated using SIS dynamics with each farm treated as a single unit. Using a top down approach, we estimate on-farm parameters associated with contraction and subsequent clearance from infection at herd level. We examine possible control strategies associated with animal movements between farms and find measures targeted at a small number of high-movement farms efficient for rapid and sustained prevalence reduction.

## Introduction

Food production for human consumption needs to become more efficient in order to respond to increasing demand for land usage and to aid in the meeting of climate change targets [1]. Endemic pathogens, such as bovine herpesvirus 1, parainfluenza 3 virus and bovine viral diarrhea virus (BVDV), have both a detrimental impact on this efficiency as well as animal welfare issues. Sustainable reduction of endemic diseases requires control measures applied across a network of farms particularly for those diseases where disease transmission is primarily associated with the movement of infected animals. These factors, along with the complexity of modeling on-farm epidemiology, suggest a top down approach to assess the impact of control measures targeting animal movement on disease burden for an endemic, economically relevant infectious disease. We here develop such an approach for the understanding of the prevalence and control of bovine viral diarrhea (BVD). Our model incorporates only key dynamics of the disease, reducing parameter estimation, and uses available data for movement and calibration where available.

BVD is a disease of cattle, endemic in many countries and caused by BVDV [2,3]. BVDV leads to a variety of health disorders including mucosal disease, immunosupression and reproductive problems. The resulting impact on economic productivity of cattle has made BVD the target of control strategies in a selection of regions including Austria, Scandinavia, Finland, Germany and Switzerland [2,4].

BVDV may be transmitted both horizontally and vertically. Infection of previously unexposed cattle results in a short transient infection followed by long lasting immunity [5]. These transiently infected cattle excrete the virus in low doses relative to a persistently infected animal (PI) [3,6]. A PI develops when a susceptible dam becomes infected in the first third of her gestation. The PI that is born remains highly infectious for its entire life. The PI may be sickly and more prone to health issues with an increased likelihood of dying younger [7]. Owing to the higher rate of viral shedding from a PI and the short period of transient infection, BVDV transmission between herds is generally accepted to occur via the direct movement of either a PI or a PI carrying dam [8].

While the general details of the BVDV epidemiology are well established, on-farm level modeling of virus transmission is complicated owing to the lack of detailed knowledge of relevant transmission parameters [9]. Numerous models have been written examining basic dynamics of the infection at the farm level [10-12]. Where possible, these have been calibrated with best available field data [11,12]. Despite the complexity of the epidemiology, certain reoccurring dynamical behaviors of farm level models may be noted. If a PI is introduced into a naive herd, transmission to the entire herd occurs relatively rapidly (within 1-8 months) [11,12]. If infection of a cow occurs during the critical portion of gestation then further PI's may be born. In this manner, BVDV may remain endemic on a farm for years. An infection free status, on a given farm, may be gained by target control or self-clearance, i.e. PI's are either moved off the farm through normal farming practices, or die off [13]. The movement of animals, some of which PIs, on and off farms implies that the process of BVDV contraction, clearance of infection and then, once the herd has become susceptible again, new infection may occur cyclically through component network farms. This results in BVDV remaining endemic to the system of farms.

There have been numerous field based studies examining the prevalence of BVD within and across communities of farms. Comparison between these studies is difficult owing to the use of different sampling methodologies and test procedures. Inherent variations in farming practices and animal trading between regions studied will also have influenced the results in these studies. A sample of surveys can give illustration of the observed variation. The proportion of the sampled population of animals that are PI's has variously been measured between 0.1-1.5 percent [6,14,15], with the number of herds containing PI's varying between 1% and 50% [16]. Large temporal fluctuations are also found in the prevalence of herds with active BVDV infection. A study of Estonia dairy herds found variously 46%, 16% and 18% suspect herds in consecutive sampling [17]. Fewer studies specifically targeting beef farms have been conducted though it is reasonable to expect similar variability.

Comparative studies indicate that network models of infection transmission are sensitive to the movement representation within a network [18,19]. Since BVDV is spread primarily through transportation of PIs, we wish to use the most detailed representation of the network, a dynamical network based upon actual movements of the animals. We choose to use the Scottish beef farm network for an implementation of our approach since both field study prevalence data and animal movement data across the network are available. Possible control strategies associated with animal movements between farms are examined. Empirical data from countries implementing BVD eradication suggests that a small, yet critical number of herd infections cannot be readily explained by animal contact between farms [20,21]. For the purpose of this study, control measures therefore aim at a considerable reduction of prevalence rather than eradication.

## Materials and methods

### Overview

Our network of farms is based upon the beef farms of Scotland. The model treats each farm as a homogeneous unit with a susceptible or infected disease status. Farm disease status is updated on a daily basis using actual daily movement data [22] of animals between the farms along with estimated probabilities of transmission and self-clearance. The two parameters of our model, i.e. disease transmission probability and farm self-clearance probability, are calibrated using a prevalence study of BVD conducted in Scotland [23]. Various control strategies to reduce endemic infection are then investigated using the calibrated model. Details of the implementation of the model and methods are discussed in more detail in the following sections. All model calculations are performed using an in-house produced C code with a time step of 1 day.

### Infection representation

We are interested in the transmission and persistence of BVDV infection across a network of farms rather than details of the transmission dynamics on the individual farms. With this in mind, the farm-scale cyclic dynamics of infection, clearance and new infection are captured using an implementation of a SIS model [24] with each farm treated as a single entity. The status of the farm is related to the presence of PIs on the farm. For the purposes of this study, a farm with at least one PI present is considered to be infected with BVDV. If a farm is in a susceptible *S *state it corresponds to no PIs present on the farm. A susceptible destination farm switches to state *I *with probability *p *when a movement from an infected departure farm occurs. *p *is associated with the probability that the transferred animal is a PI, or a PI *in utero*, and the likelihood of the infection taking hold on the farm. During calibration of the model no distinction is made between a PI and a PI *in utero*. A susceptible farm will not change status if a movement occurs from an uninfected farm.

Identification and removal of PIs clears herds of BVDV infection. Field studies also indicate that BVDV infection self-clears from an individual farm once all susceptible animals have become immune and PIs either die or are removed from the herd for non-BVD specific reasons [2,13,25]. Modeling studies of individual farms indicate that this process occurs at a rate such that the time to self-clearance approximately follows an exponential distribution from initial infection [12,26]. We model the process of clearance by assuming that an infected farm *I *changes back to a susceptible state with constant probability *μ*. The clearance half life *c*_1/2 _is then given by ln 2/*μ*. Estimated distributions of *p *and *c*_1/2 _are obtained through comparison of simulated network prevalence to observed prevalence in a cross-sectional BVD study of Scottish beef farms [23].

### Network details

**Figure 1:**
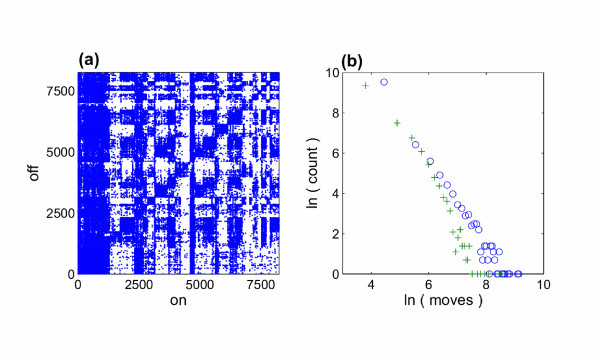
**Two static representations of the beef network in Scotland**. (a) each point represents a movement (of at least two animals) between a pair of farms. Farms are indexed via their CPH number (county, parish, holding). The higher density squares along the diagonal indicate within county movements. The higher density to the left of the plot indicates greater movement of animals into the lowest indexed county (Aberdeenshire) (b) distribution of on (circles) and off (crosses) movements from the farms. Both follow scale-free distributions with exponents 2.2 and 2.7 respectively [34].

The dynamic network of cattle movements between beef farms in Scotland is constructed based upon data provided by the Department for Environment Food and Rural Affairs, Cattle Tracing Scheme project for the period January 2007 through to December 2008 [22]. The dataset contains individual movement details for all cattle between registered premises including date of movement, animal breed and details of the premises. Holdings to be included in our network are identified as premises from which at least 20 beef breed cattle moved during the period. Where possible, the identified premises are cross checked with their registered details to ensure their validity as a farm holding. Movements between holdings via a market are incorporated in the following manner. A movement from holding B, to market, to holding C is considered as a single movement directly from holding B to holding C. Short term movements resulting in the return of an animal back to its starting holding, such as to and from a showground, are removed from the dataset [27]. Based upon this approach the resulting dynamical network [18] consisted of 7741 holdings with 544 327 movements. The network is treated as open. Movements from outside of the Scottish beef network (either geographically or from a non-beef farm) are considered movements from a single infected pool. We estimate the likelihood of an animal being a PI coming from this pool to be *p*/6 based upon reported PI prevalence [6,14,15]. This added a further 123 098 movements into the beef network. Static representations of the network are shown in Figure 1. To construct Figure 1a the holdings are firstly sorted by their CPH number (county, parish, holding) and then indexed 1 through 7741. A movement of at least two animals between a pair of holdings is indicated by a single dot in Figure 1a. The structure present in Figure 1a is mainly due to the locational information present in the 'C' (for county) component of the CPH number. Clusters of higher densities of dots are expected and correspond to either high local movements within a region/county or high movements between two regions/counties. This figure gives a novel perspective on the actual underlying local and regional connectivity of the network and could itself be used as the basis for region specific control measures. Figure 1b illustrates that the number of holdings having a certain number of either on or off movements during the period follows a scale-free distribution. It should be emphasized that Figure 1 gives static projections of the underlying network movements. The use of such static projections can lead to deficiencies in predicted disease dynamics [15], thus, in this work, we use the actual movement data as the basis of our calculations.

### Prevalence study details

A cross-sectional study of BVD herd prevalence was conducted throughout 301 Scottish beef herds visited during the period October 2006 - September 2007 [23]. The study consisted, in part, of blood sampling 10 young stock, aged between 6-16 months, from each management group. Samples were processed using an indirect ELISA test kit (Svanovir BVDV antibody ELISA, Svanova Biotech). Data was analysed at herd level using a hierarchical Bayesian finite mixing model [28] which identified three distinct cohorts of within-herd seroprevalence. Sixteen percent of herds were identified as being actively infected with BVDV and 69% of herds showed no recent exposure to BVDV. The third cohort had an intermediate seroprevalence. This was associated with exposure to BVDV within the previous five years though no current active infection.

### Parameter estimation

Parameter distributions are estimated by comparison of network prevalence found in individual simulations with the field measured prevalence outlined above. An individual simulation is run for 40 years with a random set of 15% of the 7741 farms initially infected, with the other farms initially susceptible. The 40 year simulation allows the network to settle into steady behavior, removing the dependence on initial conditions. A farm's status is incremented on a daily basis based upon any movements to the farm and BVDV clearance probability. If an animal arrives from an infected farm then the farm's status can change from *S *to *I *with probability *p*. A forty year simulation is produced by periodically repeating the base dataset of movements every two years. Each individual simulation utilized a pair of values (*p, c*_1/2_) chosen from the prior uniform distributions of [1,6] years for *c*_1/2 _and [0.005, 0.05] for *p*. For a given set of parameters, a transient of approximately 25 years is observed before the model settled into steady behavior. This method gives an estimate of the endemic prevalence that would occur assuming no changes to the number of holdings or farming methods. At the end of each simulation, the proportion of BVDV infected and herds free of BVDV, for more than 5 years, are measured across a randomly chosen sample of 301 farms. Construction of the posterior distribution is achieved by comparison of these simulated results with observed data.

An acceptable posterior distribution of parameters *p *and *c*_1/2 _is calculated using an approximate Bayesian computation rejection sampler scheme [29]. Assuming the above prior distributions, a member of the posterior distribution is determined as:

1. Sample for (*p, c*_1/2_)

2. Simulate a network dataset

3. Randomly sample 301 farms

4. If {(infected - 0.16)^2 ^+ (clear - 0.69)^2 ^≤ 0.012} accept (*p, c*_1/2_)

5. Repeat procedure

The comparative value for use with the distance metric, in step 4, is based upon the normal approximation for the 95 percentile binomial confidence interval of the observed data.

### Control strategies

The practiced systematic approach of BVD control relies upon identification of infected herds followed by detection and removal of PI's within these herds. Alongside this, biosecurity measures aimed at restricting movements of PI's, to prevent reintroduction into cleared herds, is crucial. Our model can be used to examine the impacts of a number of such systematic control strategies. PI detection and removal from herds will lead to an acceleration in the clearance rate in the model, whereas restrictions on PI movement will lead to a lowering of *p*. Here, we will examine the impact on the lowering of *p *following one of three possible levels of restriction; each of which is described in more detail below:

1. restrict all PI movements

2. restrict PI movements via markets

3. restrict all PI movements from farms of a defined size based upon the number of departures from the farm.

Simulations for each of the above are run using the posterior distributions of parameters from the previous section. Each simulation is allowed to reach a steady state and then the control strategy is introduced by setting *p *= 0 for the appropriate movements. The times taken for the herd prevalence to drop below 2% across the network is determined.

Each of the control measures relies upon accurate identification of PIs. Since PIs *in utero *are difficult to identify (unless the dam herself is a PI), the above measures will not restrict the movement of PIs *in utero*. We therefore also investigate each control strategy assuming the appropriate restriction of PIs but allowing for the movement of PIs *in utero*. We use two estimates for the proportion of transmissions (i.e. proportion of *p*) associated with movement of the unborn PIs. The ratio, 0.28, of beef animals in-calf compared to the total population size of beef animals in Scotland can be used as an upper estimate [30]. This estimate can be considered high since we do not take into account calf fatality during birthing nor any bias against the movement of older animals between holdings. A low estimate of 0.28 × 0.044 = 0.012 assumes that the fraction of these PIs *in utero *that survive birth to enter the herd is the same as the mean estimate of the number of PIs on an infected farm (See the Discussion section for further details). Both estimates ignore the possibility that the dam herself may be a PI. The PI *in utero *transmission is incorporated into the control measure simulation by reducing the probability of transmission, *p*, by either the high or low factor, for the appropriate movements.

## Results

**Figure 2:**
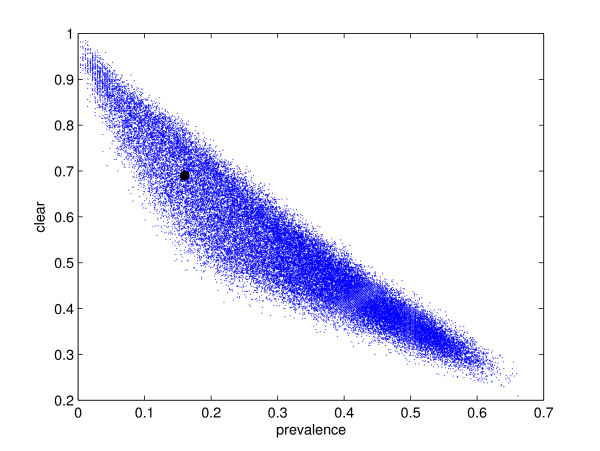
**Stochastic simulation results**. Fraction of infected farms vs. fraction of farms which have been clear for at least 5 years for a sample of stochastic simulations. Simulation parameters are selected from their prior distributions. Fractional values are calculated for a different random set of 301 farms for each simulation. The black circle shows the result of the Scottish BVD herd prevalence study. Only members of the prior distributions of parameters producing results sufficiently close to the observed value (see text for details) are chosen as members of the posterior distribution.

**Figure 3:**
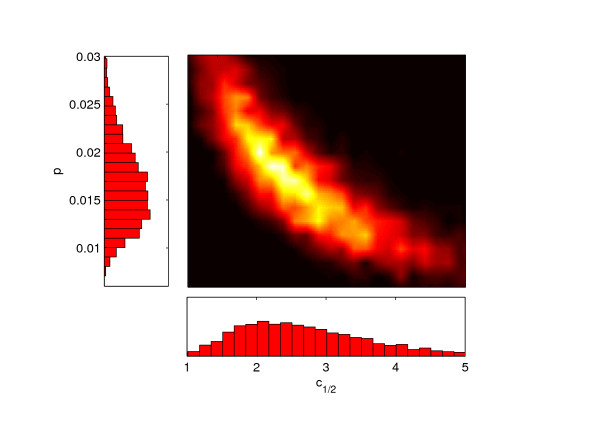
**Posterior distribution for the parameters *p *and *c*_1/2_**. Each histogram represents the frequency of occurrence of a given parameter over all values of the other parameter. On the central image, the lighter colors indicate parameter regions of higher occurrence. Times are in units of years.

A selection of simulation results produced during the model calibration stage are shown in Figure 2 along with the observed values measured during the Scottish BVD prevalence study. The two parameter posterior distributions are constructed from this plot using the Bayesian computation rejection sampler scheme. The resulting distributions based upon 100 000 iterations of this procedure are given in Figure 3.

**Figure 4:**
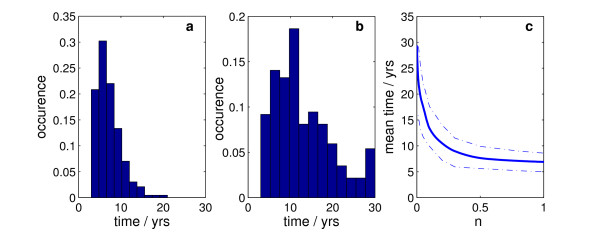
**Predicted time for BVDV herd prevalence to drop below 2% for three scenarios**. (a) no movement of PI's (b) no movement of PIs via market. Times greater than 30 years are grouped in a single bar of the histogram. (c) restrictions from farms with the higher numbers of departure moves. The x-axis corresponds to the proportion of farms, *n*, ranked according to the number of departure moves, upon which movement restrictions are placed. The solid line represents the mean time with the upper and lower lines representing the first and third quartile respectively. All simulations are run using a closed network i.e. assuming a complete ban on movement of PIs into the network.

The times taken for the herd prevalence of active BVDV infection to drop below 2% across the network are shown in Figure 4a and 4b for control strategies 1 and 2. The strategy is implemented by ranking the farms according to the number of departure movements of animals. Restriction of PI movements are then imposed on the top *n *proportion of farms. The resulting mean times to reach 2% prevalence for various values of *n *are shown in Figure 4c.

Average reduction times for each of these control strategies are shown in Table 1. Also given in this table are the corresponding times assuming that these control strategies are successfully implemented on all PIs other than PIs *in utero*.

**Table 1:** Impact of the control schemes on the average time to reach 2% herd prevalence of active BVDV infection.

Control Scheme	1	2	3(0.1)	3(0.2)	3(0.3)	3(0.5)
PIs and PIs *in utero*	7.6 ± 3.2	13.1 ± 6.2	14.0 ± 6.6	11.1 ± 5.3	9.6 ± 4.4	8.2 ± 3.9
excluding PIs *in utero*(low)	7.8 ± 3.2	13.5 ± 7.0	14.5 ± 7.1	11.4 ± 5.7	10.0 ± 4.5	8.5 ± 3.6
excluding PIs *in utero*(high)	13.6 ± 6.7	20.1 ± 8.4	21.4 ± 8.2	18.6 ± 8.4	17.6 ± 7.5	15.2 ± 7.4

Times (and standard deviations) are given for control schemes 1, 2 and 3. The number in brackets for control scheme 3 refers to the proportion of farms, *n*, to which the movement restrictions are applied. Values are given for restrictions on the movements of both PIs and PIs *in utero *as well as restrictions on the movement of PIs alone. All times are given in years.

## Discussion

To our knowledge, these distributions constitute the first estimate of effective clearance and transmission rates of BVDV infection across a network of farms. Clearly, they summarize many degrees of on-farm complexity not explicitly included in the model. We have found that the network behavior is robust to the introduction of further parameters. However, inclusion of more details in the model leads to its over parameterization with respect to the available dataset. It is of interest to see how our parameter values compare with estimates that can be made from published information. Based upon the estimate that 0.1% - 1.5% of animals across the network are PI's [6,14,15] and 16% of farms are infected [23], a range for the probability that an animal on an infected farm is a PI of 0.006 - 0.094 can be calculated. Estimates from detailed farm level models give probabilities of 0.012 (steady state value) [11] and 0.03 (time averaged value) [12]. The parameter *p *in our model is related to the probability of the infection then taking hold on the farm conditional on a PI having been moved from an infected farm. The mean value from Figure 3 (integrated across all clearance rates) is 0.016 which is in good agreement with these other estimates. To our knowledge clearance rate has never been directly measured. However, detailed farm level models of dairy holdings indicate clearance half life of ≈ 4 years [12]. The mean value for our beef network is 2.8 years.

Mean field modeling approaches to the spreading of SIS diseases across sufficiently large networks indicate the absence of an epidemic threshold [31]. This implies the disease can remain endemic for any finite transmission probability. However, empirical data gathered for BVDV shows that the relative importance of risk factors changes as prevalence decreases [32]. In the final phase of eradication 25-50% of new infections could not be explained by direct animal contacts between farms [20,21]. For example, indirect contacts between farms through contaminated equipment is likely to spread BVDV [32].

BVD eradication programs in different regions illustrate the difficulty of achieving complete eradication [33]. Following an initial significant reduction in the prevalence of BVDV infected herds, there remains a low level of infection for a considerable period of time causing new infections in previously disease free herds. Our model does not attempt to address all of the risk factors of between herd BVDV transmission. The model is targeted to investigate changes to the herd prevalence by direct contacts i.e. animal movements, and is therefore truncated at a herd prevalence of 2%. It is well suited to simulate a primary phase of reduction leading up to complete eradication; it does not attempt to include the complexity necessary to model full extinction.

The time scale of control measure 1, Figure 4a, is driven by the decay rate of the underlying exponential distribution of the BVDV infection. The mean value of 7.6 years forms a lower limit on the quickest possible reduction strategy associated purely with movement restrictions. The second movement restriction targets the livestock markets since, in principal, compliance with movement restrictions of PIs through market sites is easier to achieve than compliance for all movements between individual farms. The results of restrictions on these movements are shown in Figure 4b with a predicted mean timescale of 13.1 years. Control strategy 3 is developed based upon the special role highly connected nodes play in scale-free networks [34]. The large volume of traffic through such nodes can result in them playing an important role in BVDV transmission [31]. Targeting of the top ≈ 12% of highly connected farms is predicted to lead to the same timescale for reduction as directly targeting markets. Targeting the 50% of farms with the highest number of movements gives an average time to 2% prevalence of 8.2 years, a number comparable to the average time associated with a complete restriction on PI movements (control measure 1).

Table 1 shows the changes to the predicted average times when transmission via PI *in utero *is included in the model. Our upper estimates indicate that continued transmission via this route can almost double the amount of time taken for the network prevalence to drop below 2%. This impact can be alleviated by secondary control measures such as mandatory quarantine of new born calves from brought-in animals, until they have tested negative for BVDV.

Our model gives a first estimate of BVDV prevalence and control effectiveness across a network of farms. Future work will increase the model complexity in order to test its sensitivity to known demographic and epidemiological details. For example, owing to increased early fatality of PIs age based information can be introduced in order to account for the skew in the likelihood of PI animal transport. Similar skewing of the transmission probability by an animal's age, at the time of each movement, can also lead to improved estimates of transmission likelihood by PIs *in utero*. Another simplification we have made is to ignore effects of transmission via contact with PIs at market. Such contacts will lead to a higher prevalence of PIs *in utero *among dams moved via market and ultimately a larger impact of control measures involving market movements. A further important refinement concerns the self-clearance rate. Typically, PI numbers on a given farm are small, so that, the transfer of a single PI to another location could lead to the clearance of the infection from the departure farm i.e. the self-clearance probability has a conditional dependence upon the transmission probability. Inclusion of this dependence would be expected to lead to a slight decrease in prevalence for a given (*p c*_1/2_) pair of values. A larger value of *p *(or smaller value of *c*_1/2_) would then be required to meet the calibration criteria.

Many modeling studies of BVD have focused on detailed individual farm based models [9,11,12]. In contrast, regional control measures targeted at sustainable disease reduction generally require a systematic network wide response [13,35]. With these points in mind, we have successfully constructed a large network model of BVDV transmission using empirical movement data, validated against available prevalence information, estimated epidemiological parameters at farm level and utilized the model to evaluate control measures across the network of farms. While the presented work focuses on a single network and disease dynamic, the technique can be generally applied utilising available movement and disease information for any network and disease. By using key properties of disease dynamics and structural details of the movement network, top down estimates of hard to measure epidemiological parameters can be constructed. These estimates help to quantify transmission parameters of endemic infections instrumental for disease control across a network of units.

## Competing interests

The authors declare that they have no competing interests.

## Authors' contributions

MT constructed and co-designed the model structure. FL co-designed the model and statistical representation. FB helped with design aspects related to farm practice and disease epidemiology. All authors read and approved the final manuscript.
